# Commentary: Is There a Crucial Link Between Vitamin D Status and Inflammatory Response in Patients With COVID-19?

**DOI:** 10.3389/fimmu.2022.875973

**Published:** 2022-03-22

**Authors:** Marijn M. Speeckaert, Joris R. Delanghe

**Affiliations:** ^1^ Department of Nephrology, Ghent University Hospital, Ghent, Belgium; ^2^ Research Foundation-Flanders (FWO), Brussels, Belgium; ^3^ Department of Diagnostic Sciences, Ghent University, Ghent, Belgium

**Keywords:** vitamin D, vitamin D binding protein, COVID-19, actin, inflammation

## Introduction

With interest, we read the paper of Saponaro et al. (1), which investigated the relationship between vitamin D and a biochemical panel of inflammatory markers in patients with COVID-19. Even after controlling for age and gender, a significant inverse connection was discovered between 25OHD and interleukin (IL)-6, IL-10, tumor necrosis factor-alpha (TNF-α), C-reactive protein (CRP), and D-dimer. When compared to the other groups, patients with severe acute respiratory distress syndrome (ARDS) had a higher prevalence of hypovitaminosis D (p < 0.001), while nonsurvivor patients had lower 25-hydroxyvitamin D (25OHD) levels. We would like to emphasize the significance of vitamin D binding protein (DBP) and its polymorphism in these findings.

## Vitamin D Binding Protein: A Multifunctional Protein

Vitamin D binding protein (DBP) has a molecular weight of 52-59 kDa and is a sparsely glycosylated alpha_2_-globulin. DBP has been linked to a number of physiologically significant features. To begin with, circulating vitamin D metabolites are mostly carried bound to DBP, with albumin serving as the secondary carrier, particularly in individuals with low blood DBP concentrations (2). Humans have an extensive DBP polymorphism, with three well-known alleles (DBP1F, DBP1S, and DBP2) and a large number of race variants (> 120). Combinations of two nonsynonymous single-nucleotide polymorphisms (SNPs) in exon 11 [DBP1F (rs7041-T/rs4588-C), DBP1S (rs7041-G/rs4588-C), and DBP2 (rs7041-T/rs4588-A)] and glycosylation pattern distinguish the three prevalent phenotypic alleles (3). The median plasma concentration of 25OHD, 1,25-dihydroxyvitamin D, and DBP is determined by the DBP phenotype. Patients with the DBP1-1 phenotype have significantly higher quantities of both vitamin D metabolites and DBP than DBP2-2 carriers, whereas DBP2-1 people have intermediate levels (3). A genome-wide meta-analysis revealed that the DBP contains four SNPs that alter the concentration of 25OHD levels: rs2282679 (DBP), rs6013897 (at CYP24A1), rs10741657 (near CYP2R1), and rs12785878 (near DHCR7) (4). rs2282679 is a near-perfect proxy for rs4588: rs2282679-A is coinherited with rs4588-C, whereas rs4588-C is coinherited with rs2282679-A (5). Furthermore, the blood vitamin D concentration is genotype dependent, being highest in rs2282679-A/A participants, intermediate in rs2282679-A/C carriers, and lowest in rs2282679-C/C subjects (4). A substantial correlation between rs2282679 and blood 25OHD and DBP concentrations was found in a Mendelian randomization study. Individuals who had the C-allele at rs2282679 had lower serum DBP levels than those who carried the more prevalent A-allele (5). Changes in blood DBP concentrations and DBP polymorphisms should be regarded as possible confounders when interpreting serum total 25OHD concentrations. However, as only 1-2% of its sterol binding sites are used, DBP has been assigned a number of other metabolic activities in addition to vitamin D transport: regulation of inflammatory processes and innate immunity, scavenging actin, fatty acid binding, and bone metabolism (2).

## Discussion

When the frequency of the DBP1 allele in 55 nations was taken into account, we discovered a negative connection between the DBP1 allele frequency and COVID-19 prevalence and mortality. The relationship between the DBP1 allele frequency and a reduced prevalence and mortality due to SARSCoV2 infection might be explained in part by the putative protective effects of vitamin D and DBP (6). Furthermore, our findings were supported by a study with 517 COVID-19 patients, which found that polymorphisms in the *DBP* gene were associated with illness severity (p = 0.005). A further in-depth examination of the positive association between the Metabolism score (*DBP*, *CYP24A1*) and the severity of COVID-19 revealed that the polymorphism *DBP* rs2282679 might explain the majority of the interesting correlation found. There was a link between vitamin D polygenic risk score and blood 25OHD levels (p = 0.04). The observed association of lower 25OHD levels in nonsurvivors in the study of Saponaro et al. (1) may be influenced by DBP and its polymorphisms.

Looking at the relationship between DBP and several inflammatory markers showed that DBP synthesis is increased by IL-6, a critical driver of the hyperinflammatory condition caused by SARS-CoV-2. TNF-α causes no discernible change in DBP mRNA. Dexamethasone, on the other hand, has a positive impact on DBP release and has been demonstrated to lower mortality in critically sick patients with COVID-19 (7).

Sepsis and ARDS cause the extracellular environment and circulation to be flooded with globular actin (G-actin). G-actin buildup in the circulation causes polymerization and the development of filamentous actin (F-actin), which contributes to pulmonary microthrombi, pulmonary artery blockage, and endothelial injury (8). Lower serum DBP levels have been measured in sepsis patients compared healthy persons, with lower values in nonsurvivors versus survivors. Sepsis patients with lower serum DBP concentrations have Acute Physiology and Chronic Health Evaluation (APACHE) II and Sequential Organ Failure Assessment (SOFA) scores, indicating a negative relationship between DBP level and sepsis illness severity (9). When compared to healthy controls, the barrier integrity of primary human pulmonary microvascular endothelial cells is compromised following exposure to plasma from severe COVID-19 patients. More precisely, there is a loss of junctional VE-cadherin and cortical actin, as well as the creation of actin stress fibers and interendothelial gaps (10). Using Gene Ontology and MetaCore gene expression patterns were investigated in SARS-CoV-1 and SARS-CoV-2 infections, and revealed that actin/cytoskeleton remodeling *via* the RhoA pathway is critical for infection (11). Actin activates platelets, increasing the likelihood of thrombi formation and microcirculation blockage, a phenomena that is commonly observed in individuals with severe COVID-19 (12). The extracellular actin scavenger system is made up of two plasma proteins that serve complementary functions: gelsolin, which breaks down F-actin filaments into G-actin monomers, which are then tightly bound by DBP and transported for clearance primarily in the liver. DBP may operate as a scavenger protein in COVID-19, clearing extracellular G-actin produced from necrotic cells, which may be relevant in severe acute lung damage (13).

In conclusion, Saponaro et al. (1) conducted an intriguing investigation on the relationship between vitamin D and immune system activation during COVID-19. DBP polymorphisms influence the concentration of vitamin D metabolites and DBP. Because DBP transports vitamin D and participates in organism’s actin scavenging mechanism, we believe that future studies should focus on the link between DBP and its polymorphisms and SARSCoV2 infection.

## Author Contributions

MS wrote the first draft of the manuscript. JD edited and revised the manuscript for important content. Both authors reread, edited, and approved the final version of the manuscript for submission.

## Conflict of Interest

The authors declare that the research was conducted in the absence of any commercial or financial relationships that could be construed as a potential conflict of interest.

## Publisher’s Note

All claims expressed in this article are solely those of the authors and do not necessarily represent those of their affiliated organizations, or those of the publisher, the editors and the reviewers. Any product that may be evaluated in this article, or claim that may be made by its manufacturer, is not guaranteed or endorsed by the publisher.
